# Life cycle assessment of maize cultivation and biomass utilization in northern Thailand

**DOI:** 10.1038/s41598-020-60532-2

**Published:** 2020-02-26

**Authors:** Titaporn Supasri, Norihiro Itsubo, Shabbir H. Gheewala, Sate Sampattagul

**Affiliations:** 1 Energy Engineering Program, Faculty of Engineering, Chiang Mai University, Chiang Mai, Thailand; 2 Department of Industrial Engineering, Faculty of Engineering, Chiang Mai University, Chiang Mai, Thailand; 3 Center of Excellence on Energy, Economic, and Ecological Management (3E), Science and Technology Research Institute, Chiang Mai University, Chiang Mai, 50200 Thailand; 4 Faculty of Environmental and Information Studies, Tokyo City University, 3-3-1 Ushikubonishi, Tsuzuki, Yokohama, Kanagawa 224-8551 Japan; 5 The Joint Graduate School of Energy and Environment (JGSEE), King Mongkut’s University of Technology Thonburi, Bangkok, Thailand; 6 Center of Excellence on Energy Technology and Environment, PERDO, Ministry of Higher Education, Science, Research and Innovation, Bangkok, Thailand

**Keywords:** Environmental social sciences, Risk factors, Engineering

## Abstract

Maize, a major food source for the world, is a high-yield commodity crop, and one of five major crops in Thailand. Occupying about 33% of the Thai upland farmlands, maize farming has been growing tremendously especially in northern Thailand. However, after harvesting, open burning is widely used in order to get rid of maize cobs and husks in land preparation for the next period. The current maize farming practices have caused several problems to local communities as well as urban dwellers. The objectives of this research were: (i) to analyze the life cycle inventory of maize cultivation, maize cob pellet production and heavy fuel oil production in northern Thailand using IDEA v2.0 and ecoinvent v3.0 databases; (ii) to evaluate environmental impacts of maize cultivation, maize cob pellet production and heavy fuel oil production using A Global Scale Environmental Life Cycle Impact Assessment (LIME-3) with the results of weighting (Country-specific) based on monetary valuation of end-points. This study evaluated the life cycle environmental impacts of maize cultivation and continuing through biomass energy production from maize cob by comparing with heat production from heavy fuel oil in Mae Chaem and Chiang Dao districts in the north of Thailand by using two different databases, IDEA v2.0 and ecoinvent v3.0 with an endpoint-based life cycle impact assessment (LCIA) method (LIME-3). The system boundary of this study includes land preparation, planting, weeding, farming, harvesting, maize cob pellet production and heat production from maize cob pellet and heavy fuel oil. The units of analysis in this study are 1 kg of maize grain, 3.76E-03 MJ of biomass energy production from maize cob and 3.76E-03 MJ of heat production from heavy fuel oil, respectively. The data were obtained from field survey supplemented with the Thai National Life Cycle Inventory Database and other scientific publications. The results included the environmental impacts of maize cultivation and continuing through biomass energy production from maize cob by comparing with heat production from heavy fuel oil in Mae Chaem and Chiang Dao districts by using two different databases with LCIA method on the endpoint approaches (LIME-3). The total damage cost based on IDEA v2.0 life cycle inventory (LCI) database in Mae Chaem and Chiang Dao districts was about 4.64E-01 USD and 4.89E-01 USD, respectively. As regards ecoinvent v3.0 database, the total damage cost in Mae Chaem and Chiang Dao districts was about 5.37E-01USD and 5.99E-01 USD, respectively. It can be seen that the total damage cost using different inventory databases in Chiang Dao are slightly higher than Mae Chaem due to different input materials. The result of total cost using inventory data from ecoinvent v3.0 is slightly higher than IDEA v2.0 due to different inventory processes in each database. However, the results in this study demonstrated that the databases show similar trends in the assessment results. On the other hand, certain numerical differences between the databases at some points were found to be more substantial. The results of present study are particularly relevant to policy choices for improving or using the good practices for maize cultivation, which would reduce the environmental performance of maize production systems in the area. To address the air pollution issue from biomass open burning of agricultural residues in the study area, the government agencies in Thailand should be responsible for promoting better biomass management for the future.

## Introduction

Maize (*Zea mays* L.), a high-yield commodity crop, is a major food source for the world. In 2017, the harvested area of maize in the world was 7,209 million hectares, which decreased by 0.51% from 7,246 million hectares in crop year 2016.

**Figure 1:**
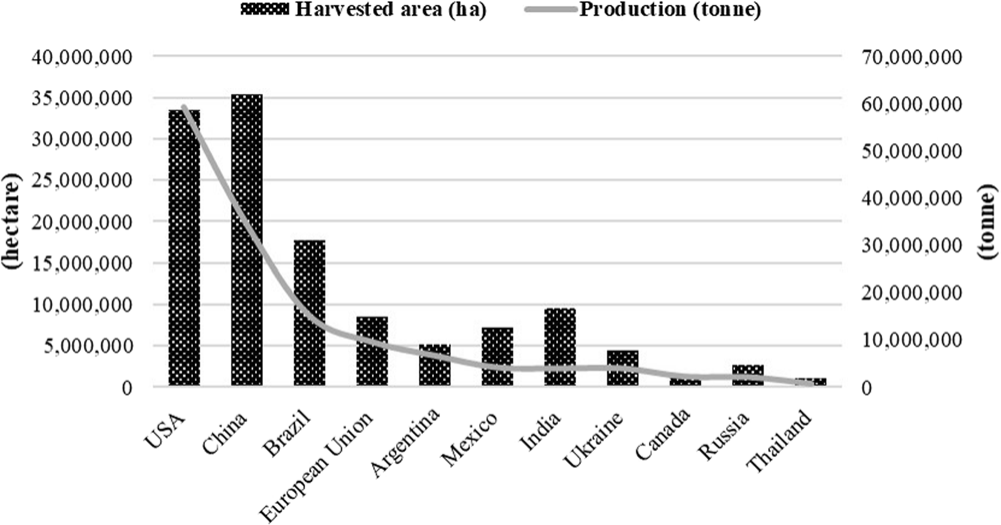
The harvested area, production and yield per hectare of maize from major countries in 2017.

Maize production was 6,527 million tonnes, which decreased by 2.87% from 6,720 million tonnes in the same period. In addition, maize yield was 5,656 kg/ha, which decreased by 2.37% from 5,794 kg/ha. USA had the highest maize production, followed by China and Brazil, respectively. Overall, maize production in most countries decreased while that in Argentina, Canada and Thailand was increasing. The harvested area, production and yield per hectare of maize from major producing countries in 2017 are shown in Fig. 1.

Maize is one of five major crops in Thailand in addition to rice, cassava, sugarcane, and rubber. It occupies a major portion (about 33%) of the Thai upland farmlands^1^. In 2017, the maize production in Thailand was 41 million tonnes, which increased from year 2016 by 1.26%. Maize production was 29 million tonnes, which increased by 7.06% from 27 million tonnes. Moreover, maize yield was 4,500 kg/ha, which increased by 5.73% from 4,256 kg/ha since it was supported by the Promotion on Dry Season Maize Production after Major Rice Crop Year 2017 Project. Moreover, maize yield increased because the amount of rainfall was enough for growth (rainy season)^2^.

**Figure 2:**
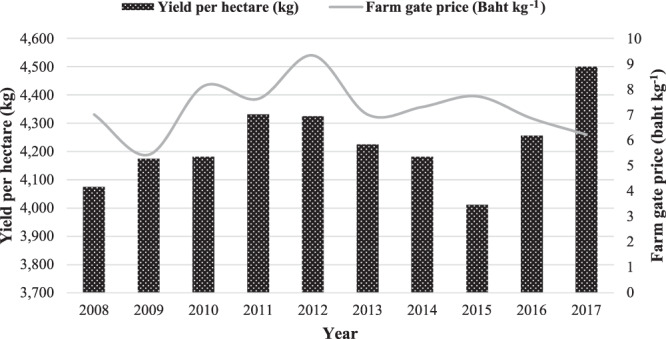
Yield per hectare and farm gate price of maize in Thailand.

Demand of maize for Thailand in 2017 was 8 million tonnes, which increased by 3.58% from 7 million tonnes in 2016. Because of the expansion of the livestock industry, demand for maize in animal feed increased. Thailand exported 0.32 million tonne of maize with a total value 72.55 million USD, which decreased from 0.6 million tonne with total value was 151.23 million USD in 2016. Maize exports and value decreased by 44.83% and 52.03%, respectively because of the increase in domestic demand of maize. As a result, maize exports were decreasing to partner countries such as the Philippines, Vietnam and China^2^. Figure 2 shows the yield per hectare and farm gate price of Thailand between 2008–2017.

In Thailand, the current maize farming practices have also caused several problems to local communities as well as urban dwellers. For example, the widespread forestland clearing has led to flooding and landslides, resulting in road accidents and crop damage. In addition, the excessive use of chemical fertilizers and pesticides has caused environmental impacts and burning crop residues after harvesting, leading to air pollution and haze which affect human health.

There are many different ways to produce biomass energy including biomass burning to generate heat in thermal systems. To compare environmental impacts of biomass energy use to continued fossil fuel use, replacing fossil fuels such as heat production from petroleum fuels with biomass energy can reduce CO_2_ emissions and avoided biomass burning after harvesting process. Therefore, it is necessary for the full utilization of biomass in order to manage the amount of biomass after harvesting and avoid biomass burning, as well as reduce the environmental impacts.

This study was conducted using the Life Cycle Assessment (LCA) methodology. LCA is a quantitative procedure to evaluate the environmental impacts within the set system boundary. Many researchers are using LCA derived information to drive business and policy decisions. The guiding principles of LCA follow the International Organization for Standardization (ISO) standards ISO 14040^3^ and ISO 14044^4^. These two ISO standards provide an overview of the steps of LCA: (1) Goal and scope definition; (2) life-cycle inventory analysis (LCI); (3) life-cycle impact assessment (LCIA); and (4) interpretation.

The objectives of this research were as follows: (i) to analyze the life cycle inventory of maize cultivation continuing through biomass energy production from maize cob by comparing with heat production from heavy fuel oil in northern Thailand using the IDEA v2.0 and ecoinvent v3.0 databases; and (ii) to evaluate environmental impacts of maize cultivation, maize cob pellet production and heat production from heavy fuel oil using LIME-3 with the results of weighting (Country-specific) based on monetary valuation of end-points.

## Literature Review

The literature review revealed that many researchers have reported the benefits of applying the LCA approach in terms of environmental management of agricultural production systems. Many studies have been conducted on environmental impacts assessment focused on maize production and biomass utilization activities. A previous study by Kim *et al*.^5^ estimated the environmental impacts for the cultivation of corn grain and corn stover in the US Corn Belt and predicted that removing corn stover from soil could reduce nitrogen emissions from soil. In 2014, Wang *et al*.^6^ examined the environmental impacts of the wheat-maize rotation system between conventional farming system and double high system in Quzhou. Boone *et al*.^7^ assessed the environmental footprint of grain maize production using the ReCiPe and CEENE methods. Recently, Fantin *et al*.^8^ assessed the environmental impacts of wheat and maize production in Italy using the ILCD approach. The major hotspot for both crops in the agricultural phase was due to fertilizers and pesticides used. Moreover, Holka *et al*.^9^ accessed two large-scale farms in the Wielkopolska region, west-central Poland during 2011–2013. The study found that the stages of crop cultivation and harvesting produced the biggest impact on acidification, eutrophication and global warming. The largest source of environmental burdens came from fertilization. Moreover, the normalized values of impact indicators showing that lifecycle environmental burdens of grain maize production were mainly associated with soil acidification. Jason *et al*.^10^ estimated GHG emissions of maize production in the US. The total climate change damage was about US$4.9 billion or 15 USD t^−1^ of maize. Also, the average health damages from air pollution were equal to 121 USD t^−1^ of harvested maize grain.

There are many impact studies focusing on biomass utilization from agricultural residues, Nilsson *et al*.^11^ analyzed the costs and energy requirements for biomass pellet production in Sweden. Song *et al*.^12^ evaluated the environmental impacts and economic value of corn straw pellet fuel in Jilin Province, China. In Thailand, open burning of agricultural residues could be avoided and the unburned residue could also substitute fossil energy. Suramaythangkoor and Gheewala^13^ evaluated the potential for heat and power generation from the rice straw that would otherwise be open burnt in Thailand. Kerdsuwan and Laohalidanond^14^ analyzed the economic aspects using corn residue for alternative energy source in order to produce power in Nakorn Sawan and Petchchaboon provinces. In the case of northern Thailand, there is a large amount of corn production as well. Therefore, Phonin *et al*.^15^ found the best clusters with minimum transportation cost for several constraints set for corn product/waste management system in northern area of Thailand using models and simulation in order to assess the total CO_2_ emissions from the waste elimination process, open burning and transforming to biomass pellet plant. Recently, Yodkhum *et al*.^16^ assessed the environmental burdens of GHG emissions, energy use, and particulate matter formation (PM_10_), from rice cultivation in Chiang Mai province, northern Thailand and compared the environmental burdens of rice straw utilization scenarios such as, open burning, incorporation into soil, and direct combustion for electricity generation.

In recent years, several life-cycle impact assessment (LCIA) studies have been published. For example, Itsubo and Inaba^17^ developed a Japanese LCIA methodology based on endpoint modeling (LIME). Then, Itsubo *et al*.^18^ developed weighting factors relevant to the Group of Twenty (G20) countries also compared the weighting factors calculated for individual countries. In addition, Yamaguchi *et al*.^19^ developed an LCIA method focused on the biodiversity in land use classification using LIME method. Murakami *et al*.^20^ estimated the willingness to pay and annual global damage cost on monetary weighting factors (MWFs) for the G20 countries. Inaba and Itsubo^21^ have developed LIME-3 in Japan using the monetary valuation method to authorize the global scale environmental impacts assessment. In order to assess the human health damage, Tang *et al*.^22^ calculated human health damage factors based on the Special Report on Emission Scenarios (SRESs) consisting of malaria, diarrhea, cardiovascular disease, malnutrition, coastal flooding, and inland flooding developed by IPCC. The results showed that the damage caused by malnutrition was the greatest, followed by diarrhea. Moreover, Tang *et al*.^23^ estimated the human health damage factors of PM_2.5_ which initiating from ten different regions of the world by using a global chemical transport model.

The present study aims at addressing the differences between the IDEA v2.0 and ecoinvent v3.0 databases using LIME-3 to analyze the environmental impact with the results of weighting (Country-specific) based on monetary valuation of endpoints. Comparison between the two databases based on the normalization results of the selected pilot study of substitution by biomass energy is conducted. The environmental impact of maize cultivation and maize cob pellet production by comparing with heat production from heavy fuel oil in northern area Thailand is then analyzed using both databases. The difference between the databases results is investigated and suggestions provided. Based on these results, suggestions on when to adopt an endpoint approach and how to interpret the different results from the two databases are provided. Finally, the limitations and recommendations of the database and approaches are discussed.

## Objectives

This study aims to analyze the life cycle inventory of maize cultivation, maize cob pellet production by comparing with heat production from heavy fuel oil in northern Thailand using IDEA v2.0 and ecoinvent v3.0 databases, as well as to evaluate environmental impacts of maize cultivation, maize cob pellet production and heat production from heavy fuel oil using LIME-3.

## Methodology

### Goal and scope of this study

**Figure 3:**
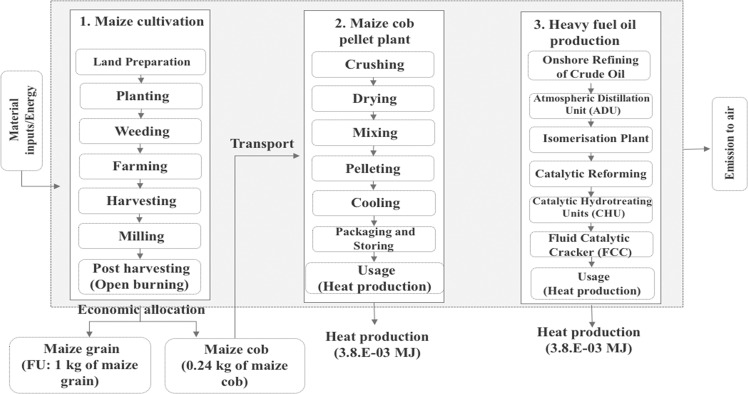
System boundary.

The goal of this study was to provide an assessment of maize cultivation, maize cob pellet production by comparing with heavy fuel oil production based on the LCA methodology. The system boundary (cradle to gate) starts from land preparation and continues through planting, weeding, and farming processes, followed by the harvesting and milling processes and post-harvesting (open burning). After the post-harvesting process, the maize cob is transported to the pellet plant for producing maize cob pellet. Moreover, this study calculated the emissions reduction by comparing with the heavy crude oil refining until the heat production from heavy oil. Economic allocation was applied to the maize grain and maize cob production, which is being sold on the market for livestock and energy applications. Maize grain and maize cob impacts were allocated using economic allocation factors according to the average market prices referring to the Office of Agricultural Economics^2^, resulting in 92% for maize grain and 8% for maize cob. The units of analysis in this study are 1 kg of maize grain and 3.76E-03 MJ of biomass energy production from maize cob and 3.76E-03 MJ heat production from heavy fuel oil, respectively. Figure 3 shows the system boundary of this study.

### Focus area

In northern Thailand, the maize area cultivated amounted to 28 million ha in 2016 with approximately, 19 million tonnes of maize grain being produce. Mae Chaem district is one of the most cultivated areas in northern region of Chiang Mai province with a cultivation area under maize of approximately 12,307 ha in 2015 while Chiang Dao district was about 5,305 ha^2^. The present study thus focuses on the important areas producing a large amount of maize in Chiang Mai, Thailand, such as, Mae Chaem and Chiang Dao districts.

**Figure 4:**
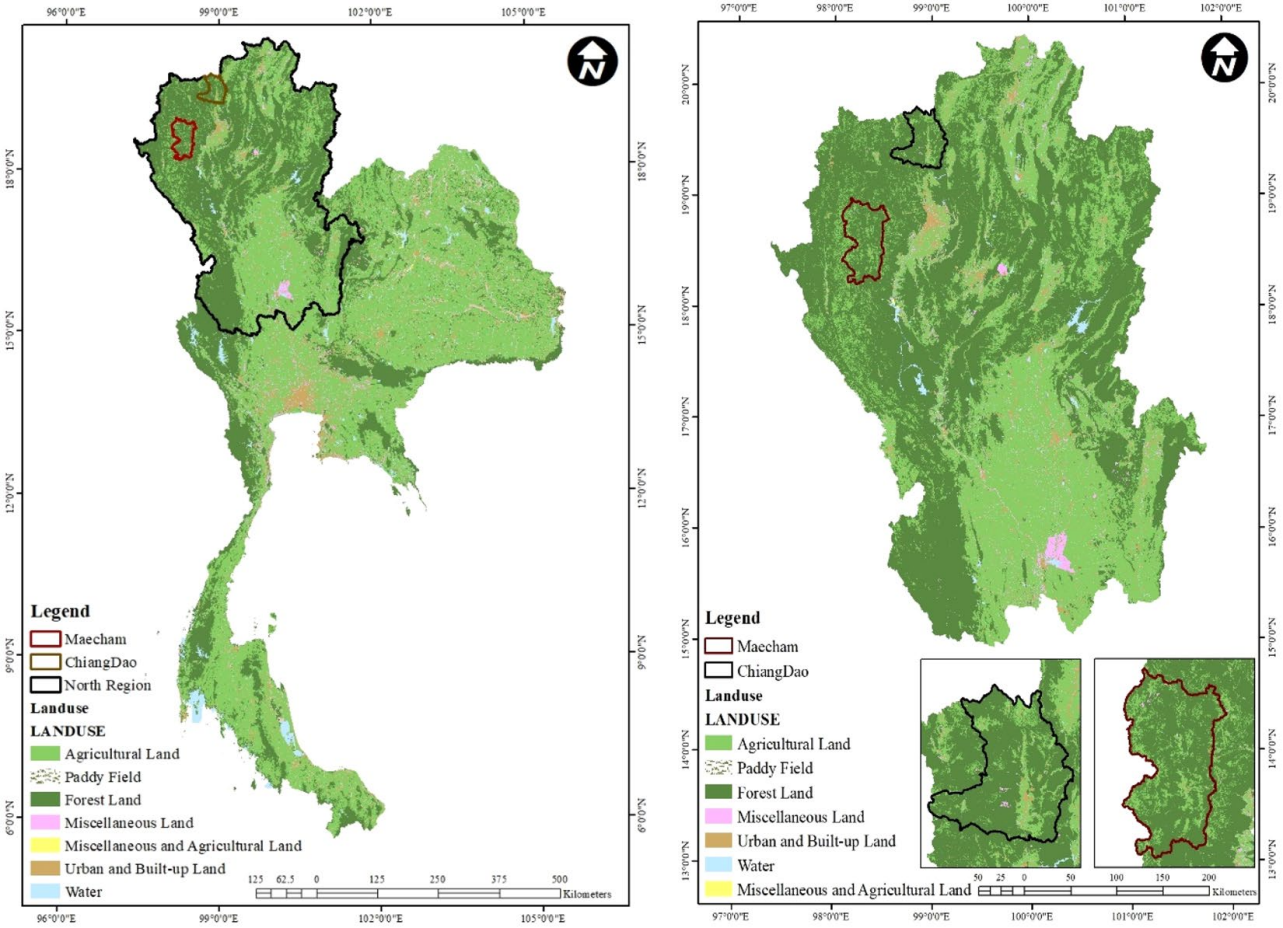
Agricultural land use map of Mae Chaem and Chiang Dao districts, northern Thailand.

The first study area, Mae Chaem, is located in the west of Chiang Mai province, in northern Thailand. Latitude and longitude of Mae Chaem coordinates are 18 °29′56″N 98 °21′43″E covering an area of 2,733.26 km^2^. Most of the terrain in the Mae Chaem district is covered by forest and upland areas. Steep mountains, piedmont alluvial plains, and plain areas make up the district at about 70%, 20%, and 10% of the total area. Forests cover about 82.08% of the total area in Mae Chaem. The next largest land use is agriculture at 16.92% of the total area. The second area, Chiang Dao, is a district located in the north of Chiang Mai. Geographically, it lies at the 19 °21′58″N 98 °57′51″E and covers an area of 1,882.1 km^2^. Most of the terrain in Chiang Dao district is covered by upland areas as well. Figure 4 shows agricultural land use map of Mae Chaem and Chiang Dao districts.

**Figure 5:**
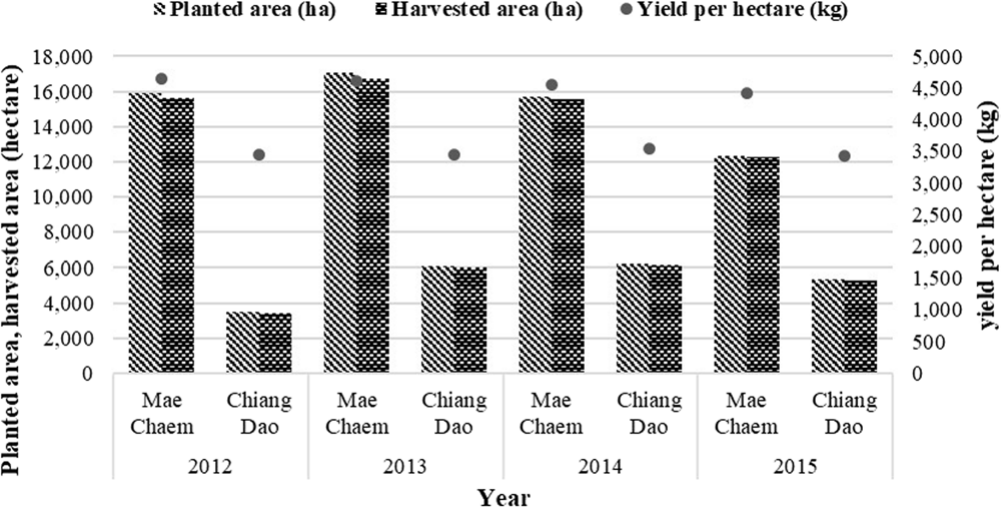
Maize: planted area, harvested area and yield per hectare of study area (2012–2015).

According to the OAE^2^, maize production in Chiang Mai in 2015 was about 603,344 tonnes; the maize residues (cobs, leaves, and stalks) were about 1,254,955 tonnes; and the maize production in Mae Chaem district was about 338,600 tonnes. The average yield per hectare was 4,400 kg while the maize residues were about 704,288 tonnes. In Chiang Dao district, maize production was about 111,906 tonnes and the average yield per hectare was 3,420 kg while the maize residues were about 232,765 tonnes. Maize farming in northern Thailand is growing tremendously. However, after harvesting processes, open burning is widely used in order to get rid of maize cobs and husks in land preparation for next period. The statistical data of planted area, harvested area, and yield per hectare of the two study areas in 2012–2015 are shown in Fig. 5.

In northern Thailand, the first crop of maize is usually grown in May. After the first rain, land preparation and sowing are started in April-May. Land preparation consists of land clearing, burning maize residue, and tillage. Land clearing and maize residues burning are often done from February to March, and tillage is mostly done in April before sowing, which is 2–7 days later; farmers start sowing the maize seeds, and they use organic fertilizers in order to accelerate the growth of maize. After 30 days, the farmers spray the weeds with herbicides. In the next 30 days, they use chemical fertilizers. The overall plantation period is 110 days. Finally, the farmers harvest their product and dry it in the field in either November or December. The farmers were observed when milling the dry maize using diesel machines. As maize residues are only partially utilized, nowadays, in the northern region, most of the maize residues (cobs, leaves, and stalks) are disposed of by open burning because it is the fastest and the most inexpensive way and it saves cost as regards to harvesting and land preparation.

Thailand has a biomass energy potential including biomass residues which can be used to produce heat energy for development of alternative energy all over the country. Especially, the north of Thailand produces a large amount of maize. According to the Department of Alternative Energy Development and Efficiency^24^, the physical properties of maize cobs consist of moisture content of 20–27%, ash content of 8% and a calorific value of 15.68 MJ/kg. The market price of maize pellets is about 3,000–4,000 baht/tonne (about 100–130 USD/tonne). Maize cob pellets are used in small-scale boilers for domestic thermal production that can replace conventional oil (heavy fuel oil) or gas boilers for industries that require large amounts of heat such as, tobacco curing plants, dried longan factory and maize grain drying. Generally, the processes of maize cob pellet production in Thailand are quite simple with very few stages which are crushing of raw material, drying, pelletizing, cooling, and packaging and storing^25^.

### Data collection and inventory data

The primary data in this study were obtained directly by face-to-face questionnaires in totally 100 rai (16 hectare) of maize production area in the study areas in 2016. The data include raw materials for maize production such as fertilizers, herbicides, including diesel fuel which is used in agricultural machinery and transportation. The calculations and assumptions are identifiable based on the secondary data in the existing databases in SimaPro, such as IDEA v2.0^26^, ecoinvent v3.0^27^, the Thai national database, the EMEP/EEA air pollutant emission inventory^28^ and IPCC^29^ guidelines. For the maize production stage, the inputs include maize seeds, fertilizers, pesticides, diesel, and transportation. The system outputs contain all of the products and co-products as well as pollutants emitted to the environment. The inputs and outputs of the various stages in maize production are shown in Tables 1 and 2.

**Table 1:** Inputs of the various stages in maize production.

Focus area	Maize production Stage	Input	Quantity	Unit
**Mae Chaem**	Land preparation	—	—	—
	Planting	Maize seed	18.75	kg ha^−1^
		Organic fertilizer	1,250	kg ha^−1^
		NPK (15:15:15) Fertilizer	312.50	kg ha^−1^
	Weeding	Atrazine	2.19	kg ha^−1^
		Alachlor	3.13	kg ha^−1^
	Farming	NPK (15:15:15) Fertilizer	93.75	kg ha^−1^
		Urea (46-0-0)	312.50	kg ha^−1^
	Harvesting and Milling	Diesel	6.25	L ha^−1^
	Post harvesting (Open burning)	Diesel	3.13	L ha^−1^
**Chiang Dao**	Land preparation	Diesel	18.75	L ha^−1^
	Planting	Maize seed	18.75	kg ha^−1^
		Diesel	18.75	L ha^−1^
	Weeding	Paraquat dichloride	25	L ha^−1^
	Farming	Urea (46-0-0)	312.50	kg ha^−1^
		Paraquat dichloride	12.50	kg ha^−1^
	Harvesting and Milling	Diesel	62.50	L ha^−1^
		Fertilizer bag	62.50	kg ha^−1^
	Post harvesting (Open burning)	Diesel	6.25	L ha^−1^

**Table 2:** Outputs of the various stages in maize production.

Focus area	Output	Quantity	Unit
Mae Chaem	**Main product**		
	Maize yield	4,400	kg ha^−1^
	**Co-product**		
	Maize residue (leaves, and stalks)	8,119	kg ha^−1^
	Maize residue (cob)	1,059	kg ha^−1^
	**Emissions**		
	Airborne emissions from diesel^28^		
	Fertilizers emissions^29^		
	Herbicides emissions (air and soil)^29^		
Chiang Dao	**Main product**		
	Maize yield	3,419	kg ha^−1^
	**Co-product**		
	Maize residue (leaves, and stalks)	6,288	kg ha^−1^
	Maize residue (cob)	820	kg ha^−1^
	**Emissions**		
	Airborne emissions from diesel^28^		
	Fertilizers emissions^29^		
	Herbicides emissions (air and soil)^29^		

For the maize cob pellet production stage in this study, the inputs include only electricity consumption for operating the machines. The production output is 1 tonne/hour of maize cob pellet with a net calorific value of about 15.68 MJ/kg. Totally, heat production from this pellet plant is about 15,680 MJ/hour. Table 3 shows the inputs and outputs of maize cob pellet and heat production for 1 tonne/hour. As regards to heavy fuel oil production, the inputs started with the onshore refining of crude oil, Atmospheric Distillation Unit, isomerization plant, catalytic reforming, Catalytic Hydro treating Units, Fluid Catalytic Cracker and heat production from heavy fuel oil.

**Table 3:** Inputs of maize cob pellet and heat production.

Maize cob pellet production Stage	Input	Quantity	Unit
Crushing	Electricity	2.2	kWh/t
Drying	Electricity	7.5	kWh/t
Pelleting	Electricity	2.2	kWh/t
Cooling	Electricity	1.1	kWh/t
Packaging and Storing	Electricity	2.2	kWh/t
Usage (heat)	Maize cob pellet	1	tonne

### Life cycle impact assessment

**Figure 6:**
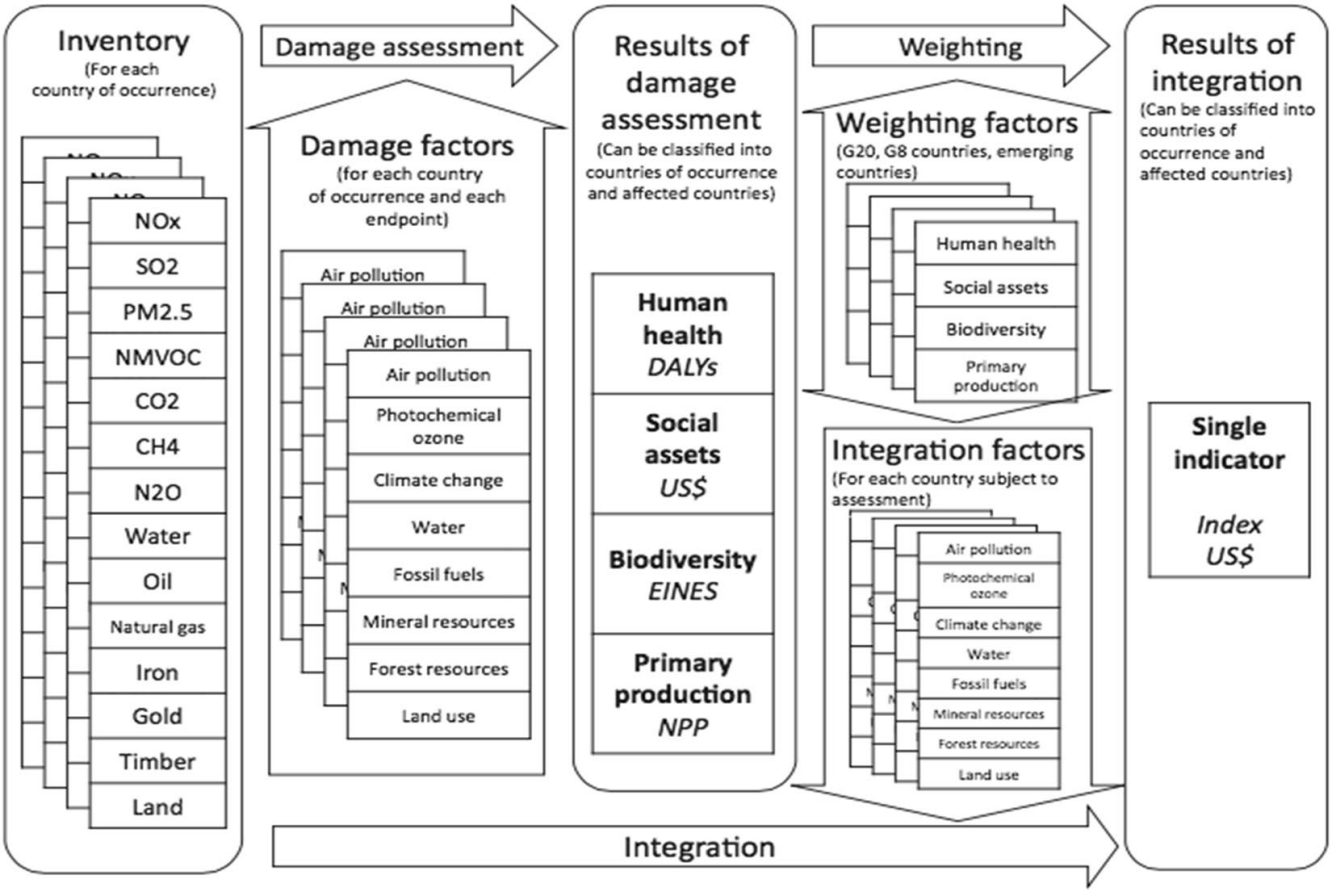
Conceptual figure of LIME-3.

LIME-3 was developed in 2016 in order to be used in the entire world. Nine impact categories of characterization which included climate change, air pollution, photochemical oxidants creation, water consumption, land use, mineral resource consumption, fossil fuel consumption, forest resource consumption, and solid waste were analyzed and four endpoints which comprise human health, social assets, biodiversity, and primary production. The aggregate analysis for weighting factors was conducted in all G20 countries. This study used country-specific weighting factors for Thailand to calculate damage cost with interest rate of about 5% as the results of weighting based on monetary valuation of end-points (USD). The conceptual figure of LIME-3 is shown in Fig. 6.

## Results and Discussion

### Results of life cycle inventory analysis

The study collected the LCI data from the IDEA v2.0, ecoinvent v3.0 databases, focusing on the inventory related to impact assessment on climate change, air pollution, photochemical oxidants creation, water consumption, land use, mineral resource consumption, fossil fuel consumption and forest resource consumption such as, CO_2_, SO_2_, NO_x_, PM_2.5_, Oil, Coal, Natural gas, Water, Land transformation and Land occupation. The life cycle inventory databases of IDEA v2.0 and ecoinvent v3.0 are shown in Table 4.

**Table 4:** Life cycle inventory database of IDEA v2.0 and ecoinvent v3.0.

IDEA v2.0 database									
Inventory	Unit	LCI of maize cultivation (FU: 1 kg of maize grain) (1)		LCI of maize cob pellet production including heat production (FU: 3.76E-03 MJ of heat production) (2)		LCI of heavy fuel oil including heat production (FU: 3.76E-03 MJ of heat production) (3)		Total LCI (4) = (1) + (2) − (3)	
		Mae Chaem	Chiang Dao	Mae Chaem	Chiang Dao	Mae Chaem	Chiang Dao	Mae Chaem	Chiang Dao
CO_2_	kg	2.27E-01	2.93E-01	7.16E-05	7.16E-05	3.02E-04	3.02E-04	2.27E-01	2.92E-01
SO_2_	kg	2.77E-04	2.36E-04	2.93E-08	2.93E-08	2.51E-07	2.51E-07	2.77E-04	2.35E-04
NO_x_	kg	3.71E-03	4.54E-03	3.52E-08	3.52E-08	1.57E-07	1.57E-07	3.71E-03	4.54E-03
PM_2.5_	kg	1.15E-02	1.18E-02	7.24E-10	7.24E-10	1.11E-08	1.11E-08	1.15E-02	1.18E-02
Oil	kg	2.86E-02	6.05E-02	1.48E-06	1.48E-06	9.27E-05	9.27E-05	2.85E-02	6.04E-02
Coal	kg	9.23E-03	5.75E-03	9.66E-06	9.66E-06	2.28E-09	2.28E-09	9.24E-03	5.76E-03
Natural gas	kg	5.93E-02	6.49E-02	1.69E-05	1.69E-05	1.66E-06	1.66E-06	5.93E-02	6.50E-02
Water	m^3^	2.25E-03	1.25E-03	4.29E-11	4.29E-11	1.35E-12	1.35E-12	2.25E-03	1.25E-03
Land transformation	m^2^	8.53E-05	1.10E-04	2.01E-13	2.01E-13	2.64E-15	2.64E-15	8.53E-05	1.10E-04
Land occupation	m^2^y	1.20E-03	1.49E-04	3.47E-11	3.47E-11	1.16E-12	1.16E-12	1.20E-03	1.49E-04
**ecoinvent v3.0 database**									
**Inventory**	**Unit**	**LCI of maize cultivation (FU: 1 kg of maize grain) (1)**		**LCI of maize cob pellet production including heat production (FU: 3.76E-03 MJ of heat production) (2)**		**LCI of heavy fuel oil including heat production (FU: 3.76E-03 MJ of heat production) (3)**		**Total LCI (4) = (1)** **+** **(2)** **−** **(3)**	
		**Mae Chaem**	**Chiang Dao**	**Mae Chaem**	**Chiang Dao**	**Mae Chaem**	**Chiang Dao**	**Mae Chaem**	**Chiang Dao**
CO_2_	kg	3.38E-01	5.47E-01	1.02E-04	1.02E-04	6.95E-04	6.94E-04	3.37E-01	5.47E-01
SO_2_	kg	6.72E-04	1.32E-03	3.10E-08	3.10E-08	5.33E-06	5.33E-06	6.67E-04	1.32E-03
NO_x_	kg	3.83E-03	4.78E-03	8.04E-08	8.04E-08	1.22E-06	1.21E-06	3.83E-03	4.78E-03
PM_2.5_	kg	1.15E-02	1.19E-02	3.20E-09	3.20E-09	3.87E-08	3.87E-08	1.15E-02	1.19E-02
Oil	kg	3.39E-02	6.81E-02	3.81E-07	3.81E-07	2.18E-04	2.18E-04	3.37E-02	6.79E-02
Coal	kg	1.54E-02	1.88E-02	1.92E-06	1.92E-06	8.66E-06	8.66E-06	1.53E-02	1.88E-02
Natural gas	kg	4.86E-02	6.29E-02	2.94E-06	2.94E-06	8.16E-06	8.15E-06	4.86E-02	6.29E-02
Water	m^3^	5.96E-11	7.39E-11	1.89E-15	1.89E-15	1.30E-14	1.29E-14	5.95E-11	7.39E-11
Land transformation	m^2^	1.24E-02	1.70E-02	3.03E-08	3.03E-08	1.58E-08	1.58E-08	1.24E-02	1.70E-02
Land occupation	m^2^y	6.87E-03	2.54E-02	2.85E-08	2.85E-08	1.36E-08	1.36E-08	6.87E-03	2.54E-02

The results show the difference in values when using the two LCI databases. The latest versions of the two databases at the time the research was carried out were used. In the IDEA v2.0 database, primary inventory data are estimated based on each statistical classification to assure high exhaustiveness and detailed inventory data of various products, and activities are calculated in more detailed classification classes from literature, theoretical figures and process data of factory. However, this database covers only Japanese industrial activities. It is necessary to collect inventory data of foreign countries and to consider with imported products. On the other hand, the ecoinvent database comprises of the LCI data covering all economic activities which describes an activity at a unit process level. The LCI results of ecoinvent datasets are used for identifying environmentally preferable goods or services. Therefore, the database should be considering the relevance and completeness of the data for the specific assessment. However, the results in this study demonstrated that the databases show similar trends in the assessment results. On the other hand, certain numerical differences between the databases at some points were found to be more substantial.

### Results of weighting calculation using the IDEA v2.0 database

**Figure 7:**
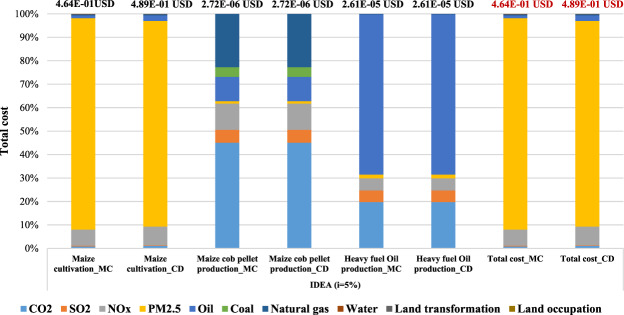
The weighting result of case study using IDEA v2.0 database (i = 5%).

The results of the weighting calculation for the main step of maize cultivation phase, maize cob pellet production and heavy fuel oil production are divided in two different areas using the IDEA v2.0 LCI database and presented in Fig. 7 as percentage contributions to the damage cost. In Mae Chaem district, maize cultivation (1 kg of maize grain) produced the biggest damage cost which is approximately 90% in PM_2.5_, and the cost is about 4.18E-01 USD. The second damage cost occurred in NO_x_ and is about 3.21E-02 USD, or approximately 7%. The results present the main cause of the contribution which is from the biomass burning in the post harvesting process. As regards to maize cob pellet production (3.76E-03 MJ of heat production), the biggest damage cost occurred in CO_2_ and is higher than 40%, and the cost is about 1.22E-06 USD, followed by natural gas which is about 6.19E-07 USD or 23%. Regarding the heavy fuel oil production (3.76E-03 MJ of heat production), the largest damage cost contributed by oil is almost 70%, and the cost is about 1.78E-05 USD followed by CO_2_ which is about 5.16E-06 USD or 20%. Finally, the results of total damage cost are the highest in PM_2.5_ at approximately 90%, followed by NO_x_ which is about 7%. The results of Chiang Dao district are similar to those of the Mae Chaem district. The results show that the biggest damage cost in maize cultivation, maize cob pellet production and heavy fuel oil production occurred in PM_2.5_, CO_2_ and oil which is about 4.29E-01 USD or 88%, 1.22E-06 USD which is approximately 45%, and 1.78E-05 USD which is approximately 70%, respectively.

Regarding the total damage cost based on the IDEA v2.0 LCI database, Mae Chaem and Chiang Dao districts contributed a total damage cost of about 4.64E-01 USD and 4.89E-01 USD, respectively. The main contributions from the maize cultivation are about 4.64E-01 USD and 4.89.E-01 USD, respectively. The hotspot total damage cost is the highest in PM_2.5_ or approximately 90%, followed by NO_x_ which is about 7% at Mae Chaem district and the total damage cost is the highest in PM_2.5_ or approximately 88%, followed by NO_x_ which is about 8% in Chiang Dao district. Although the results of the two areas were similar, the total damage cost in Chiang Dao is slightly higher than Mae Chaem, resulting in the total damage costs depending on the various input materials in each process.

**Figure 8:**
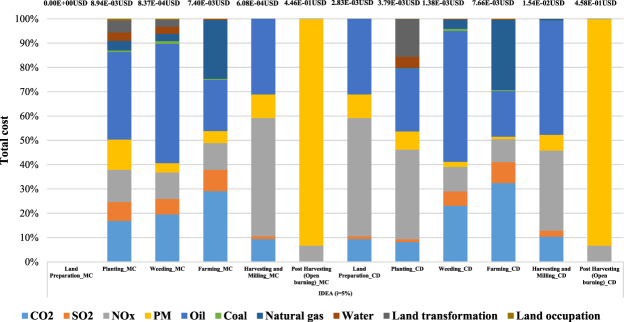
Total damage cost contribution of maize cultivation using IDEA v2.0 database (i = 5%).

Regarding the main contribution of total damage cost based on the IDEA v2.0 LCI database, the results present the largest contribution which is from the maize cultivation phase (1 kg of maize grain) occurred in PM_2.5_ in both areas which is approximately 90% due to biomass burning in the post harvesting process. The total damage cost of PM_2.5_ in post harvesting process by biomass burning is about 4.16E-01 USD at Mae Chaem and 4.27E-01 USD at Chiang Dao. Figure 8 shows the total damage cost contribution in maize cultivation using IDEA v2.0 database.

### Results of weighting calculation using the ecoinvent v3.0 database

Referring to the results of weighting calculation using the ecoinvent v3.0 LCI database, maize cultivation (1 kg of maize grain) in Mae Chaem produced the biggest damage cost which is almost 80% in PM_2.5_, and the costs is about 4.19E-01 USD and the largest damage cost in Chiang Dao also occurred for PM_2.5_ at approximately 72%, which is about 4.32E-01 USD. The main results of PM_2.5_ in both areas are from the biomass burning in the post harvesting process. The next contribution is land transformation from the current practice of maize cultivation which would be move to new crop every year. The damage cost of land transformation is 6.7E-02 USD (12%) and 9.2E-02 USD (15%) respectively. As regards to maize cob pellet production (3.76E-03 MJ of heat production) in Mae Chaem and Chiang Dao, the highest damage cost contributed is from CO_2_ which contributes approximately 60%. The cost is about 1.8E-06 USD, followed by NO_x_ which is about 7.0E-07 USD (almost 25%) in both areas. As regards to heavy fuel oil production (3.76E-03 MJ of heat production) in Mae Chaem and Chiang Dao, oil has the highest contribution which is about 4.2E-05 USD (45%) and the second contribution is SO_2_ which is about 2.7E-05 USD (approximately 30%) in both areas.

Regarding the ecoinvent v3.0 LCI database, the total damage cost of Mae Chaem is about 5.37E-01 USD. The highest damage cost from the maize cultivation is about 5.37E-01 USD. Also, with Chiang Dao district, the highest damage cost from the maize cultivation is about 5.98E-01 USD and the total damage cost is about 5.98E-01 USD. As regards the total damage cost of two areas, PM_2.5_ has the highest at approximately 78% and 72% in Mae Chaem and Chiang Dao, respectively, followed by land transformations which are about 13% and 15%, respectively. In summary, these results are similar to those using the IDEA database; Chiang Dao district produced a higher damage cost than Mae Chaem district resulting from different input materials.

**Figure 9:**
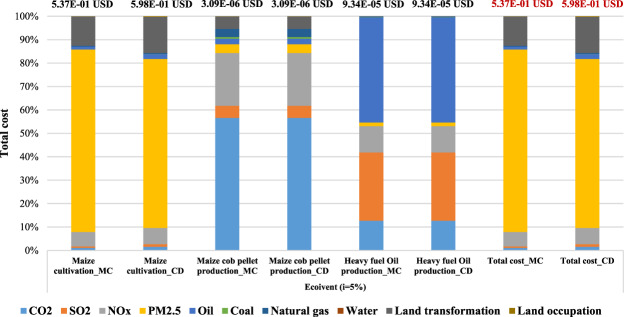
The weighting results of case study using ecoinvent v3.0 database (i = 5%).

Overall, the results of total cost using different inventory databases showed that ecoinvent v3.0 is slightly higher than IDEA v2.0 caused by different inventory processes and data collection, as well as the system boundary in each database. Figure 9 shows the percentage contribution to the damage cost for the case study using ecoinvent v3.0 database.

**Figure 10:**
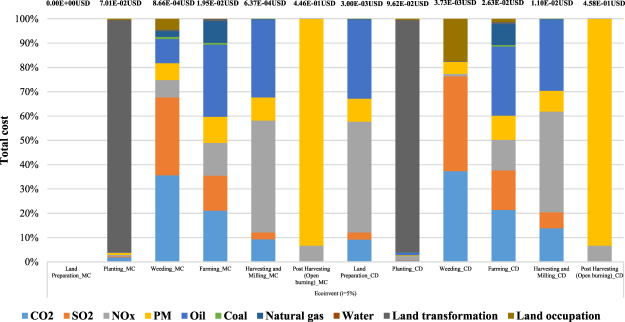
Total damage cost contribution of maize cultivation using ecoinvent v3.0 database (i = 5%).

Regarding the main contribution of total damage cost based on the ecoinvent v3.0 LCI database, the results present the largest contribution which is also from the maize cultivation phase (1 kg of maize grain) occurred in PM_2.5_ in both areas which is approximately between 72–80% due to biomass burning in the post harvesting process. The total damage cost of PM_2.5_ in post harvesting process by biomass burning is about 4.16E-01 USD at Mae Chaem and 4.27E-01 USD at Chiang Dao. The total damage cost contribution in maize cultivation using the ecoinvent v3.0 LCI database is shown in Fig. 10.

### Comparison with other studies

**Figure 11:**
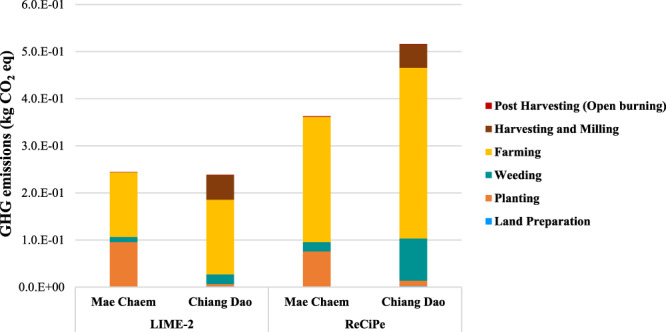
GHG emissions of maize cultivation (1 kg of maize grain).

Finally, the results of this study were compared to those obtained from published LCAs on maize cultivation^5,6,9,30^ since 2009–2017, resulting in GHG emissions of maize cultivation for 1 kg of maize grain. Nevertheless, all the analyzed studies differ in terms of considered system boundaries, the methodological assumptions, the impact assessment methods and the respective environmental indicators. Therefore, a comprehensive comparison was not always possible although the findings of this study generally correspond to the main outcomes of the other LCAs. As regards to the maize cultivation phase in this study, we assessed the environmental impact for the potential for global warming impact category using LIME-2^17^ and ReCiPe method^31^ to compare mid-point impact categories of global warming. The highest contribution to global warming impact category occurred in the farming process which is from fertilizer used in both two areas. Moreover, the results of GHG emissions by using ReCiPe method are slightly higher than LIME-2 method (approximately 20–30%). The results of GHG emissions of maize cultivation for 1 kg of maize grain are shown in Fig. 11.

**Figure 12:**
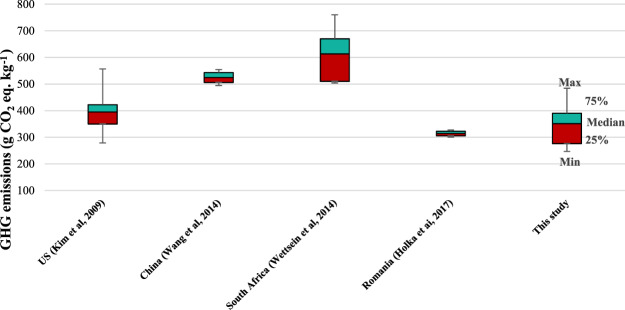
Total GHG emissions of 1 kg of maize grain compared with other studies.

As regards to the result of maize cultivation in 1 kg of maize grain for comparing with other studies, the results of this study per 1 kg of maize grain gave a total GWP impact of approximately 351.2 g CO_2_eq. This result is presented by box plot to display the five-number summary of a set of data including the minimum, first quartile (25%), median, third quartile (75%), and maximum. Figure 12 presents a comparison of the results of maize cultivation in 1 kg of maize grain with other studies using box plot.

Regarding the environmental impacts of maize production in this study, not only the highest contribution in global warming impact occurred in the farming process from the excessive use of chemical fertilizers and pesticides, there was also an important impact causing air pollution in post harvesting process from the biomass open burning. In order to reduce the environmental problem of maize production systems in the study area, encouraging farmers to plant alternative crops can lead to sustainable agriculture by providing the farmers with alternatives suitable for their area. Avoiding biomass burning after harvesting process, the agricultural residues are processed to pellets (biomass energy production) widely used for direct burning as fuel for industries that require large amounts of heat, such as tobacco curing plants or dried longan factory. Also making organic fertilizer in order to manage agricultural residues should be considered to solve the problem. Furthermore, maize straw incorporation directly into field has been promoted as a source of organic matter and a way to increase soil quality. Therefore, full incorporation of maize residues could significantly improve soil fertility and maintain crop yields, also reducing the GHG emissions for the focus area.

Increasing land transformation due to the shifting cultivation practice has led to the invasion problem of reserved forest areas by encroaching farmers in northern Thailand. In this study, the areas have been facing the problem of air pollution over the past decade, particularly during the dry season from February to May which is the same period of maize cultivation. The mainly burned areas are forest and agricultural areas. Therefore, air pollution issue has to be discussed for the best practice/policy/strategy and action plan to improve the knowledge on maize cultivation and air quality management. In addition, the support for biomass management should be promoted by the government agencies in Thailand.

As regards to the comparison of replacing fossil fuels as heat production system with biomass energy as a case study of biomass pellet, the results presented that biomass fuel can significantly reduce the total GHG emissions. This study assessed the environmental impacts savings that biomass feedstocks which could be used in heat production in the northern, Thailand offer compared to fossil fuels (generally heavy fuel oil). High yielding crops (about 3–4 tonnes/ha) have a high biomass energy from maize cob pellet conversion efficiency to replace current heavy fuel oil consumption. The analysis has resulted in the following issues which are related to the environmental impacts of maize cultivation, such as GHG emissions and air pollution, with a separate policy best practice/policy/strategy and action plan in order to manage the biomass after harvesting and avoid biomass burning.

## Conclusion

This study presents an LCA study of maize cultivation and continuing through biomass energy production from maize cob by comparing with heat production from heavy fuel oil in northern Thailand using the IDEA v2.0 and ecoinvent v3.0 databases. The results according to the LIME-3 method showed the major hotspots for both areas in the nine impact categories and calculate damage cost with interest rate is about 5% as the results of weighting based on monetary valuation of end-points (USD).

Based on the IDEA v2.0 database, the largest contribution for maize cultivation (1 kg of maize grain) for both areas was from PM_2.5_ at approximately 90%, the next highest being from NO_x_ at approximately 7% which was from the biomass burning in the post harvesting process. Regarding the maize cob pellet production (3.76E-03 MJ of heat production), the biggest damage cost in both areas occurred due to CO_2_ from electricity consumption contributing more than 40%. The largest damage cost of the heavy fuel oil production (3.76E-03 MJ of heat production) was contributed by oil at almost 70%. Finally, the total damage cost based on IDEA v2.0 LCI database in Mae Chaem and Chiang Dao districts was about 4.64E-01 USD and 4.89E-01 USD, respectively.

As regards ecoinvent v3.0 database, the largest damage cost of the two areas from maize cultivation (1 kg of maize grain) was in post harvesting (open burning) with PM_2.5_ contributing almost 80%. The next damage cost was land transformation at approximately 15% which from the current practice of maize cultivation would be moved to the new crop every year. The largest hotpot in maize cob pellet production (3.76E-03 MJ of heat production) was from CO_2_ reaching 60% mainly from electricity consumption in machines. The biggest contributor of the heavy fuel oil production (3.76E-03 MJ of heat production) which from oil at about 45%. The total damage cost based on ecoinvent v3.0 in Mae Chaem and Chiang Dao districts is about 5.37E-01USD and 5.99E-01 USD, respectively.

Overall results of total damage cost using different inventory database for both system boundaries in Chiang Dao are slightly higher than Mae Chaem due to different input materials. For the result of total cost using different inventory databases, ecoinvent v3.0 is slightly higher than IDEA v2.0 caused by different inventory processes in each database. However, the results in this study demonstrated that the databases show similar trends in the assessment results.

The results of the present study are particularly relevant to support policy choices for improving or using the good practice for maize cultivation in the north of Thailand, which would reduce the environmental problem/GHG emissions and air pollution of maize production systems. The findings could also be used to further enhance the policy/strategy and action plan to improve biomass management in the agricultural area and air quality management in the north of Thailand for maximum efficiency and sustainability in the future.
