# Author Correction: Life cycle assessment of maize cultivation and biomass utilization in northern Thailand

**DOI:** 10.1038/s41598-021-03592-2

**Published:** 2021-12-16

**Authors:** Titaporn Supasri, Norihiro Itsubo, Shabbir H. Gheewala, Sate Sampattagul

**Affiliations:** 1 Energy Engineering Program, Faculty of Engineering, Chiang Mai University, Chiang Mai, Thailand; 2 Department of Industrial Engineering, Faculty of Engineering, Chiang Mai University, Chiang Mai, Thailand; 3 Center of Excellence On Energy, Economic, and Ecological Management (3E), Science and Technology Research Institute, Chiang Mai University, Chiang Mai, 50200 Thailand; 4 Faculty of Environmental and Information Studies, Tokyo City University, 3-3-1 Ushikubonishi, Tsuzuki, Yokohama, Kanagawa 224-8551 Japan; 5 The Joint Graduate School of Energy and Environment (JGSEE), King Mongkut’s University of Technology Thonburi, Bangkok, Thailand; 6 Center of Excellence On Energy Technology and Environment, PERDO, Ministry of Higher Education, Science, Research and Innovation, Bangkok, Thailand

Correction to: *Scientific Reports* 10.1038/s41598-020-60532-2, published online 26 February 2020

In the original version of this Article errors were made during the conversion from rai to hectares. As a result, in the Introduction,

“In 2017, the maize harvested area in Thailand was 41 million hectares, which increased from year 2016 by 1.26%.”

now reads:

“In 2017, the maize production in Thailand was 41 million tonnes, which increased from year 2016 by 1.26%.”

In addition, in the Methodology section, under the subheading “Focus area”,

“Mae Chaem district is one of the most cultivated areas in northern region of Chiang Mai province with a cultivation area under maize of approximately 480,756 ha in 2015 while Chiang Dao district was about 207,213 ha^2^.”

now reads:

“Mae Chaem district is one of the most cultivated areas in northern region of Chiang Mai province with a cultivation area under maize of approximately 12,307 ha in 2015 while Chiang Dao district was about 5305 ha^2^.”

Furthermore, in Table 1, the “Quantity” for Focus area “Mae Chaem”; “Harvesting and Milling” was incorrect. The incorrect and correct value appears below.

Incorrect:

**Table:** 

Focus area	Maize production stage	Input	Quantity	Unit
Mae Chaem	Harvesting and milling	Diesel	5.21	L ha^−1^

Correct:

**Table:** 

Focus area	Maize production stage	Input	Quantity	Unit
Mae Chaem	Harvesting and milling	Diesel	6.25	L ha^−1^

In Table 2, the quantities for Focus area “Chiang Dao”; “Maize yield” and “Maize residue (leaves, and stalks)” were incorrect. The incorrect and correct values appear below.

Incorrect:

**Table:** 

Focus area	Output	Quantity	Unit
Chiang Dao	**Main product**		
	Maize yield	3,420	kg ha^−1^
	**Co-product**		
	Maize residue (leaves, and stalks)	6,290	kg ha^−1^

Correct:

**Table:** 

Focus area	Output	Quantity	Unit
Chiang Dao	**Main product**		
	Maize yield	3,419	kg ha^−1^
	**Co-product**		
	Maize residue (leaves, and stalks)	6,288	kg ha^−1^

Finally, in Figure 1 the number of harvested area (hectare) and production (tonne) was corrected. In addition, three different variables were plotted in Figure 1, but the axes did not reflect all three variables. As a result, “Yield per hectare (kg)” has been removed for clarity. In Figure 5, the number of planted area (hectare) and harvested area (hectare) was corrected.

The original Figures 1 and 5 and accompanying legends appear below.

**Figure 1:**
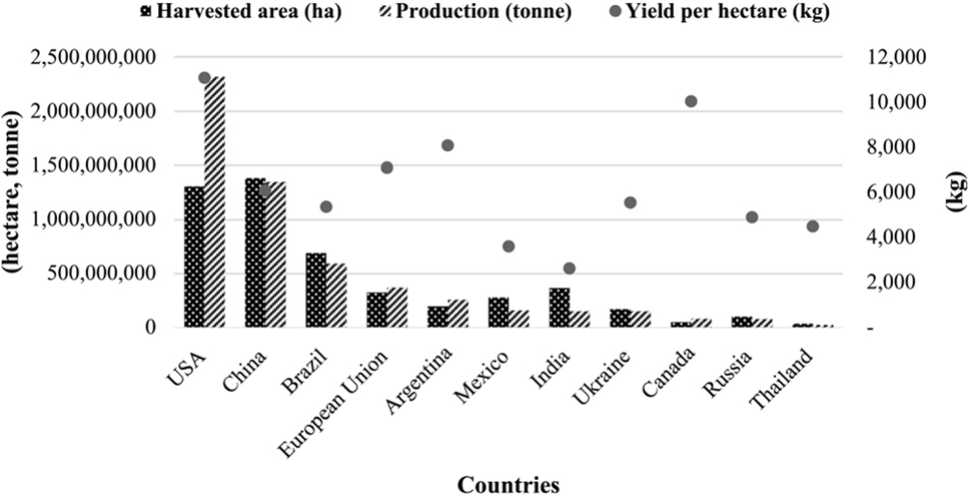
The harvested area, production and yield per hectare of maize from major countries in 2017.

**Figure 5:**
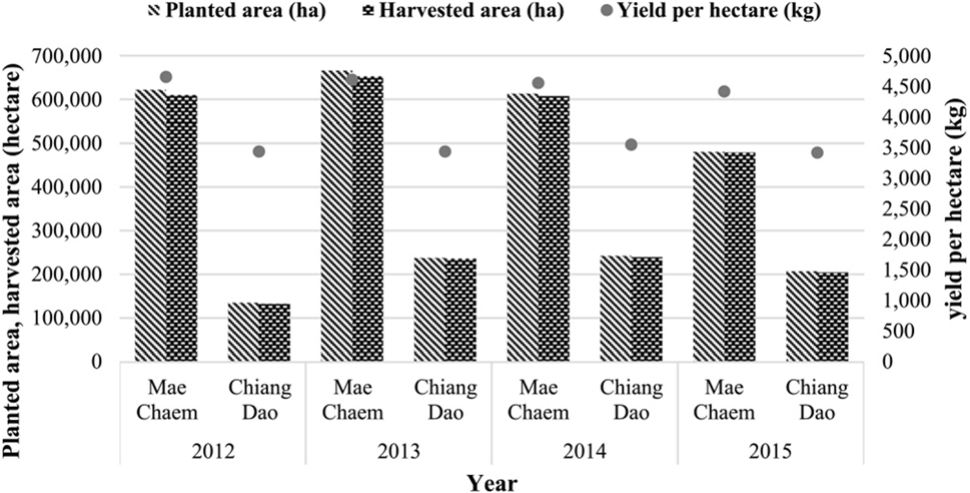
Maize: planted area, harvested area and yield per hectare of study area (2012–2015).

The original Article has been corrected.

